# Microscopic Simulating the Impact of Cruising for Parking on Traffic Efficiency and Emission with Parking-and-Visit Test Data

**DOI:** 10.3390/ijerph19159127

**Published:** 2022-07-26

**Authors:** Xinliu Sui, Xiaofei Ye, Tao Wang, Xingchen Yan, Jun Chen, Bin Ran

**Affiliations:** 1 Faculty of Maritime and Transportation, Ningbo University, Fenghua Road 818#, Ningbo 315211, China; suixinliu123@163.com; 2 School of Architecture and Transportation, Guilin University of Electronic Technology, Lingjinji Road 1#, Guilin 541004, China; wangtao_seu@163.com; 3 College of Automobile and Traffic Engineering, Nanjing Forestry University, Longpan Road 159#, Nanjing 210037, China; xingchenyan.acad@gmail.com; 4 School of Transportation, Southeast University, Si Pai Lou 2#, Nanjing 210096, China; chenjun@seu.edu.cn; 5 Department of Civil and Environmental Engineering, University of Wisconsin–Madison, 1415 Engineering Drive, Madison, WI 53706, USA; bran@seu.edu.cn

**Keywords:** cruising for parking, VISSIM, traffic efficiency, traffic emission, parking layout optimization

## Abstract

Cruising for parking creates a moving queue of cars that are waiting for vacated parking spaces, but no one can see how many cruisers are in the queue because they are mixed with normal cars. In order to mitigate the influence of cruising for parking on normal cars, the simulation framework based on VISSIM was proposed for reproducing the cruising vehicles and normal traffic flows. The car-following model of cruising vehicles was calibrated by the GPS and video data. The scenarios under different cruising ratios were analyzed to evaluate the influence of cruising for parking on traffic efficiency and emissions. Finally, the layout optimization changing the parking locations and positions of entrance-exit gates were discussed to mitigate the negative effect. The results indicated that cruising for parking deteriorates the traffic congestion and emissions on the road sections, intersections and network. The closer distances the intersections and sections are to the parking lot, the greater the negative impact is. But the negative effect after the 30% proportion on traffic performance only illustrates the slight deterioration, because the carrying capacity of the network is reached. The research results provide a quantitative method for the hidden contribution of cruising for parking on traffic congestion and emissions.

## 1. Introduction

The problem of parking difficulty limits the sustainable development of the Central Business District (CBD) of most cities. Due to the disequilibrium of high parking demand and low supply, vehicles are compelled to search for a vacated parking space until one car departs. These vehicles cruising for parking usually drive at low speeds, change lanes frequently and swerve hurriedly. They assemble a moving and invisible queue to deteriorate traffic congestion and pollution in the CBD of the city. From Donald Shoup’s observation in Los Angeles [1], the cruising vehicles mixing in the normal traffic flow accounted for 30% and spent an average of 8 min to find a curbside lot. Nearly 1.61 million vehicle miles traveled (VMT) per year were wasted by cruising for parking, which consumed extra 47,000 gallons of gasoline, and emitted 730 tons of carbon dioxide (CO_2_). Obviously, cruising for parking worsens traffic congestion and exacerbates air pollution.

Moreover, with the emergence of autonomous vehicles (AVs), the self-parking capability is likely to choose the lowest cost parking space around the trip destinations or not park at all. The extra floating trips of cruising for parking are also generated, while AVs search the cheaper peripheral parking space; or find the free on-street parking; or only cruise around the road to wait for the next trip. Consequently, any improvements in efficiency, energy consumption and parking requirements might be compensated by the increase in VMT that AVs cruising for the free parking and waiting for the next passenger would bring. Whether the future age of AVs or the current era of human-driven vehicles, the quantitative influence of cruising for parking must first be evaluated reliably.

Because the cruisers are usually hidden behind the normal traffic flow, it is difficult and costly to observe their influences directly. Previous parking studies mainly focused on alternative methods to measure and identify cruising behaviors without empirical data. And the cruising behaviors of AVs are also difficult to be verified in real-world traffic situations. Therefore, the quantitative effects of cruising for parking have been rarely modeled and studied, particularly with experimental data. In order to mitigate the influence of cruising parking on traffic congestions and emissions, the floating car experiment in park and visit tests of GPS trajectories were designed to collect the cruising behavior and calibrate the parameters of the cruising car-following model, and the videotape of traffic flows was used to identify the volume of cruising cars and the traffic status of normal traffic flow, simultaneously. Then the microscopic modeling of cruising for parking under the actual road network environment was constructed to estimate the quantitative impacts on traffic efficiency and emission. The sensitivity analysis was adopted for the different proportions of cruising traffic flow. Finally, the choices of parking locations and the changing positions of parking entrance-exit gates were discussed to optimize and minimize the negative effect of cruising for parking. The findings could explain how cruising cars add to the normal traffic that is already congested, and quantifies the worse situation of their influence.

The contributions of the study were as follows: (1) We quantified how cruising affects traffic efficiency. (2) We measured how cruising aggravates traffic emissions. (3) We showed how the locations and gates of parking lots affect traffic efficiency and emissions.

## 2. Literature Review

Cruising has aroused the extensive concern of scholars. For example, Shoup [1] found that the average cruising time of vehicles was about 8 min, and about 30% of vehicles in the traffic flow were cruising vehicles. The On-Street Parking Management [2] mentioned that when the parking was close to saturation (the occupancy rate is more than 85%), it was difficult for drivers to find parking spaces and cruise for parking. At the same time, the cruising traffic in the saturated parking congestion area usually accounts for more than 30% of the traffic flow. Arnott et al. [3,4,5] and Van Ommeren [6] both proved that cruising behavior aggravated traffic congestion. Zhu et al. [7] found that between 9 and 56 percent of the traffic was cruising for parking, and the average search time was about 6.03 min. The low-speed, volume, high acceleration frequency and lane-change times of cruising cars had a negative effect on shortening on the travel time of the normal traffic flow.

Some scholars have used different methods to study the effect of cruising. Mannini et al. [8] used floating car data (FCD) of the test vehicle to identify the cruising vehicles and model their cruising time. Geroliminis [9] proposed a model of cruising. This model considered the dynamics of different types of vehicles when driving or cruising to the destination. The results showed that cruising for parking affected all vehicles in the road network. Levin et al. [10] developed algorithms to provide optimal guidance to individual drivers on where to search for or reserve parking and how to navigate the traffic while searching for parking to alleviate the congestion caused by searching for parking. Dowling et al. [11] introduced methods for analyzing a special class of networks of finite capacity queues. They applied this model to estimate the proportion of drivers cruising in the neighborhood of Belltown, Seattle, WA, USA. Using occupancy approximated by parking transaction data, the percentage of cruising for curbside parking was estimated by comparing the rate of drivers unable to find parking to bulk through traffic measurement data. Du et al. [12] presented a method of mathematical programs with equilibrium constraints to determine the optimal sites of street parking facilities in a working area where drivers travel through or cruise for parking spaces in the morning commute. The optimal sites of street parking facilities incur the shortest queuing delay in the capacitated network. The results showed that the appropriate allocation should avoid parking spaces at the curbs of arterials where travelers drive for traversing the area and the streets intersecting the arterial. Van Ommeren et al. [13] introduced a novel methodology to estimate the marginal external cruising time (and thus cost) across time and space: the marginal external cost of parking. This methodology makes it easy for cities to evaluate the optimality of their current parking fees and parking supply from a welfare perspective. Ding et al. [14] built a parking-cruising behavior model on the road be considered the influence of time value, the results showed that the search time of non-work trips was longer than that of work trips. Arnott [4] analyzed the change in parking search time from namely walking distance and parking space supply and demand and established a model with parking space occupancy rate as the optimal target. According to the research, activities in a specific period would aggravate the generation of parking cruising behavior. Lee et al. [15] argued that perceived costs are one of the major drivers of the cruising behavior and drivers were not well informed on parking costs, even when they claim to be familiar with these costs.

Some scholars have explored the factors affecting cruising for parking and the mutual relations among these factors. Liu & Geroliminis [16] used the Macroscopic Fundamental Diagram to find that commuters’ cruising distance and time for parking are higher with decreasing curbside parking vacancies during the morning peak. Qin et al. [17] explored car drivers’ cruising behavior and location choice for curb parking in areas with insufficient parking space. Moreover, the results showed that the closer to the destination car drivers are, the more likely they choose to park on the curb. The results provided a better understanding of cruising behavior for parking and recommendations for reducing cruising for parking. Van Ommeren & Russo [18] found that the average cruising time for parking is about 36 s when the price of curb parking is the same as off-street parking. Meanwhile, the cruising time for parking increases with travel time and parking duration.

Some scholars have also studied the optimization scheme to reduce AVs’ cruising for parking. Zhao et al. [19] presented a centralized parking dispatch approach to optimize the distribution of floating AVs and provided regional route guidance. Moreover, the results proved that parking dispatch and regional route guidance of AVs were effective in reducing intense cruising for parking traffic. Shafiei et al. [20] investigated the dynamics of travelers shifting to private-owned AVs from other transport modes and their negative impacts on road traffic congestion. The results indicated that the distance-based pricing scheme could effectively limit the usage of private AVs and reduce traffic congestion, especially in the city center and peripheral suburbs. Yet another study suggested that time-based congestion pricing was the key factor to hinder AVs from cruising that exacerbating congestion [21].

Some scholars have studied the impact of cruising for parking on emissions. Based on a group of studies mentioned in Shoup [1], the cruising time to find a vacant parking space was between 3.5 and 14 min. Searching for empty parking spaces leads to cruising and frequent stops which increases CO_2_ emissions. Paidi et al. [22] found that excess CO_2_ emissions and non-optimal were mainly observed during visitor peak hours when there were limited or no empty parking spaces in the region of interest, which also leads to excess cruising. A facility location and allocation scheme was engaged to determine the optimal number and locations of public parking lots in Aydin’s study. Additionally, three objectives were considered: minimizing total facility establishment and operational cost, minimizing the average distance traveled, and minimizing CO_2_ emissions within the system [23]. Shen et al. [24] proposed an optimal parking site selection scheme to alleviate CO_2_ emissions of the traffic flows for green urban road networks. Through the creative dynamic traffic zone programming, a constrained model was set up to assess the impact of potential public parking locations on road traffic emissions. Čuljković [25] found an adequate parking price can eliminate cruising for parking and provide significant effects on fuel savings and pollutant emissions. Ye et al. [26] found that the traffic emission have an impact on the parking choice behavior of shared autonomous vehicles, and it is more significant in short-term parking than long-term parking, which means that travelers pay more attention to traffic emission in short-term parking than in long-term parking. Previous parking studies have mainly focused on the substitute methods to measure and identify cruising behaviors without empirical data. Moreover, the cruising behaviors of AVs were evaluated by the simulation framework and could not be verified in real-world traffic situations. In this paper, the experimental data were collected for modeling the impact of cruising for parking on traffic congestion and emissions.

## 3. Data Collection

**Figure 1:**
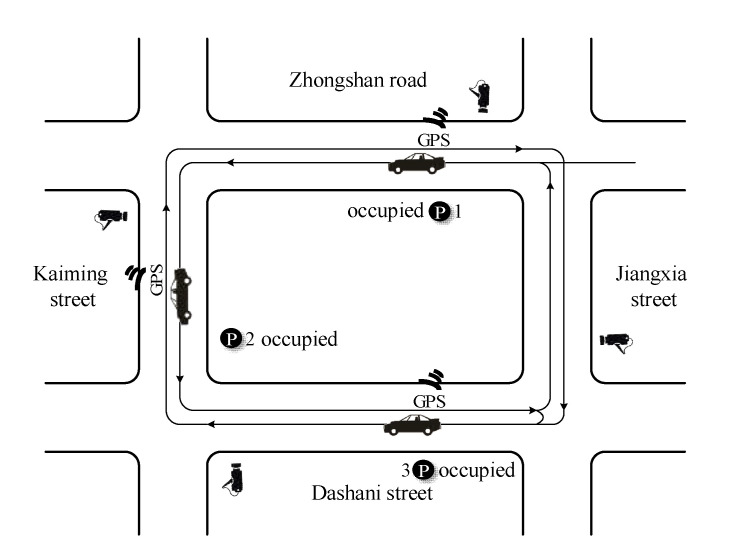
Investigation plan.

Park-and-visit tests with GPS and cameras were applied to capture the data of cruising for parking. GPS equipment was used to record the cruiser’s driving trajectory (latitude and longitude), velocity, acceleration and deceleration, lane-changes, number form stops and other data in the statistical intervals of 5 s. The cruising time of the experimental car for every park-and-visit test was also calculated by GPS data. As an analogy to the floating car experiment, two inside cameras were equipped in the cruising vehicle for recording the number of vehicles in the forward and reverse direction flow. As shown in Figure 1, other cameras were set up at the observation points to measure the volume of cruising cars and the traffic status of normal cars. The cruising process is as follows: a moderate distance is chosen for experimental testing to ensure that the plan is feasible [27]. Then the driver independently selects a destination and first choice parking lot within the survey area and informs the investigator to record the whole trip from the starting point to the destination without interference. If the first choice parking is occupied, the experimental vehicle starts to cruise for a vacated parking space around the study area. Until the cruiser finds an available parking space, the park and visit test could be finished once and drive out of the study area to start a new test.

Because high parking demand in the Tianyi Square CBD of Ningbo city creates a high probability of parking cruising behavior, the data collection was conducted in this area during the peak and off-peak hours (16:00–19:00). The 456 experiments were recorded in 14 days. The average cruising time of vehicles was 6.03 min, the average velocity of cruising cars was 13.53 km/h, the average acceleration was 0.25 m/s^2^, the average acceleration times was 27.41, and the average lane-change times was 4.79. The speed-changes and lane-changes of cruising vehicles are significantly higher than normal traffic flow. More characteristic parameters of cruising vehicles were also obtained in Table 1.

**Figure 2:**
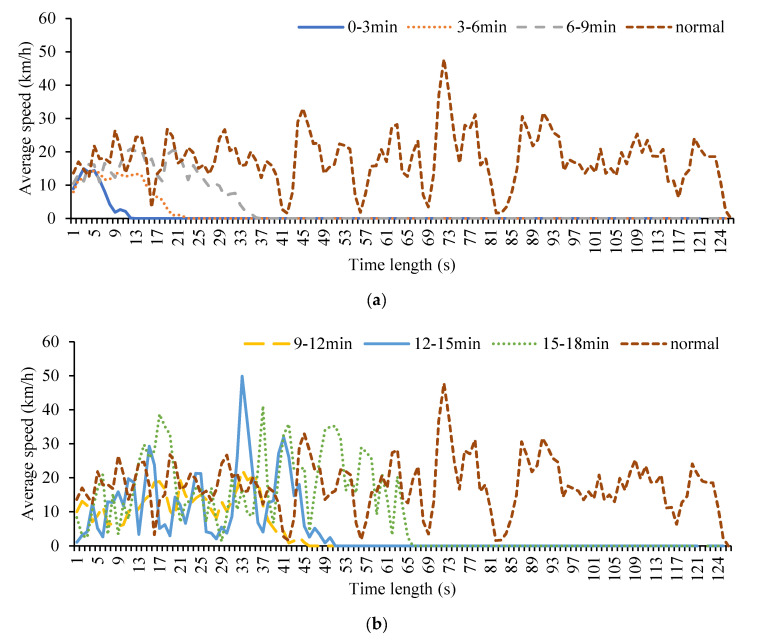

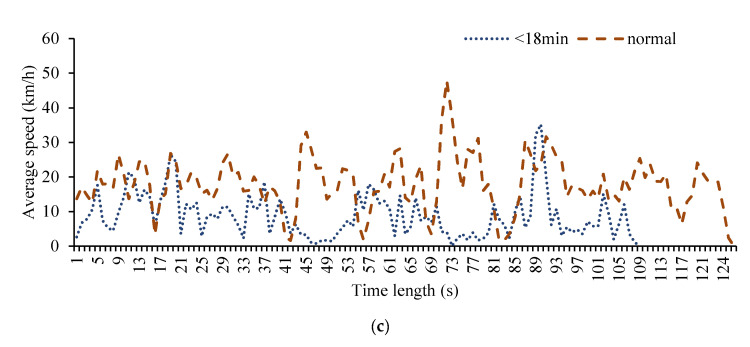
Comparing the average speed of cruising vehicles with normal vehicles speed under different cruising times. (**a**) Cruising time ranging from 0 to 9 min. (**b**) Cruising time ranging from 9 to 18 min. (**c**) Cruising time less than 18 min.

From the camera datasets, the average velocity of normal traffic flow is obviously higher than the cruisers, which is 22.13 km/h. The traffic flow of cruising for parking accounts for 9 and 56 percent. As shown in Figure 2, the shorter the searching time by a cruising car, the more frequent speed changes were. After it was more than 12 min, the speed change presented periodic oscillations. Cruising for parking would result in a remarkable drop in the overall speed.

## 4. Microscopic Modeling Cruising for Parking

### 4.1. Parameter Calibration for Cruising Traffic Flow Model

**Figure 3:**
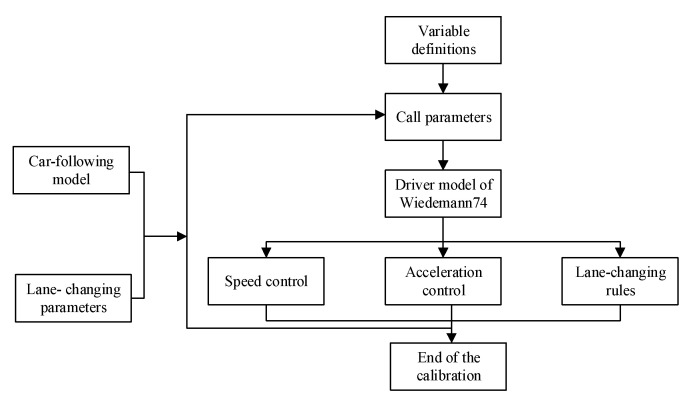
The setting process of driving behavior parameters of cruising vehicles.

Compared with the normal passing vehicle, the driving behaviors of cruising vehicles are reflected by the reaction time of cruiser, distracted time, safety clearance, minimum headway and lane-change behavior. The setting process is designed for calibrating the driving behavior parameter of cruising vehicle in Figure 3.

As a classical car following model, Wiedemann74 was chosen for simulating the cruising traffic flow. It could be formulated as:

(1) d=ax+bx

(2) $$bx=(bxadd+bxmult\times z)\times \sqrt{v}$$

where *ax* denotes the expected stopping distance; *bx_add_* represents the expected car-following distance and the coefficient; *bx_cult_* is the expected car-following distance multiplication coefficient; *z* defines as the random factor between [0,1], the average value is 0.5, the normal distribution of the standard 0.15. *v* represents the speed of the car.

The parameters of the Widemann74 car-following model were calibrated by GPS and video data, which combined the operating conditions of the eight intersections and related road sections with GPS trajectories. Then the actual situations of traffic rules, driver habits, cruiser behaviors and other aspects, referring to relevant literature [28,29] were also considered to optimize the range of the corresponding parameters in the Widemann74 car-following model, as shown in Table 2.

### 4.2. Simulation Framework Based on VISSIM

**Figure 4:**
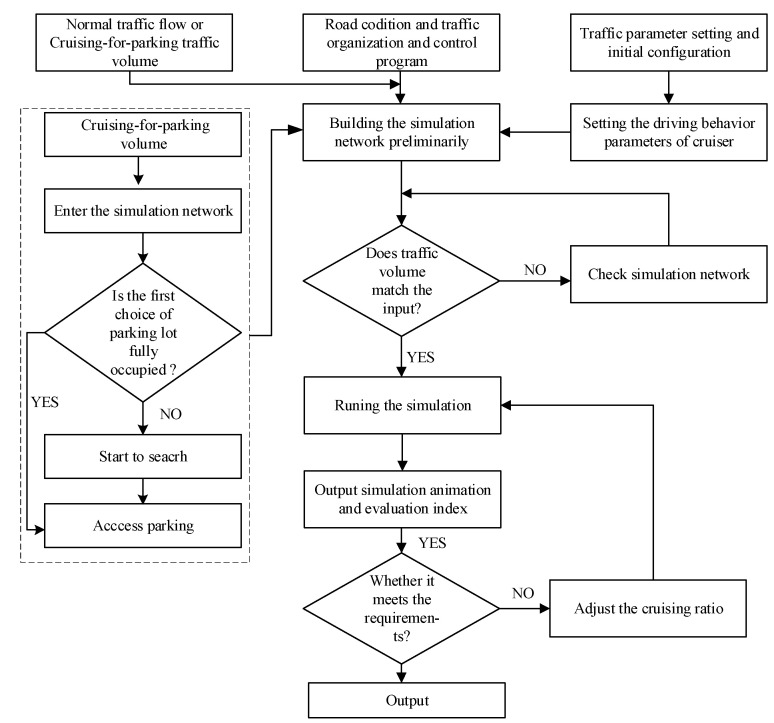
Framework of VISSIM simulation.

As shown in Figure 4, the road condition, normal traffic flow parameters, signal control, parking facilities and other relative factors in the survey area are simulated in the VISSIM software. The Widemann74 car-following model of the cruiser is also mixed in the traffic flow model to assess the interactive influence of cruising for parking. In order to generate sufficient cruising behaviors, parking choice events are integrated into VISSIM through the second development COM interface. On the left of Figure 4, the cruising vehicle typically experiences four parking-related events, which contain entering the area, determining if the first choice parking lot is saturated, starting to search for vacant parking space, and accessing the vacant parking berth.

**Figure 5:**
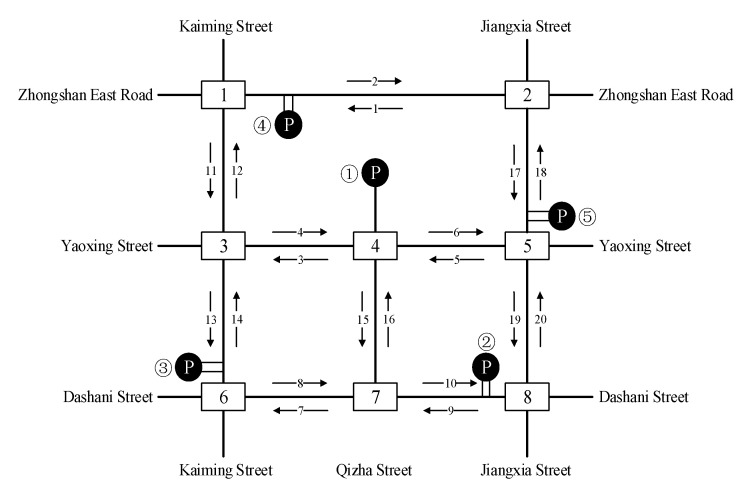
Simulated road traffic network.

As shown in Figure 5, the area covers the complete road network of Tianyi Square and contains five parking lots, nine main roads and eight signalized intersections. Their names are numbered below to clarify the positions of the five representative parking lots in the road network. The real-world traffic volume, speed and signal control from the practical investigation data are chosen as the control group in the simulation experiment. Then the cruising behavior under different parking demand-supply patterns, parking resource utilization and traffic conditions are added into the simulation framework to examine the resulting traffic efficiency and emission.

## 5. Empirical Results

First, the results of the control groups are simulated and validated by the real-world traffic conditions within the error of 5%. Then the volume of cruising vehicles is input into the simulation framework incrementally, while other variables of traffic conditions are at their real-world values. Finally, the mixed traffic flow status and emissions with the cruisers are assessed by comparing the control group and different cruising for parking scenarios for the road segments, intersections, and networks.

### 5.1. Influence of Cruising for Parking Behavior on Traffic Efficiency

#### 5.1.1. Influence of Different Cruising Ratios on Traffic Flow at the Intersections

Under the conditions of 0% (basic scenario), 5%, 10%, 15%, 30%, 50% cruising traffic flows, five common indicators of intersection level of service (LOS) including average queue length, maximum queue length, average traffic volume, average vehicle delay and average number form stops at the intersection are chosen in Table 3.

**Figure 6:**
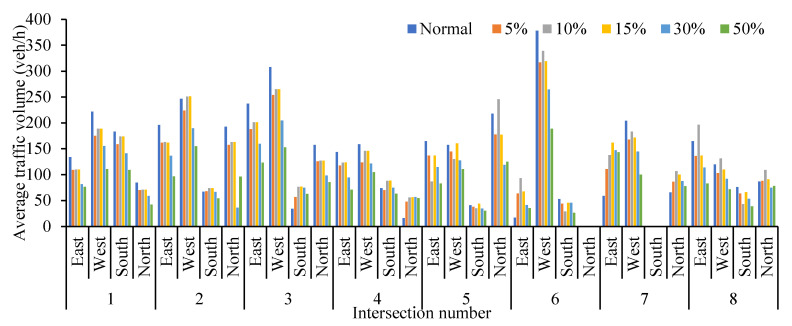
Comparison of average traffic volumes at different intersections.

According to the comparison of four measures of effectiveness (MOEs) at eight intersections with different cruising ratios, traffic congestion caused by cruising for parking behavior results in a decrease in average traffic volume at each intersection with the proportion of cruising vehicles increases, as shown in Figure 6. Most of the vehicles cruising for parking back and forth around the parking lot form a relatively fixed search path, so the average traffic volume of south import of intersections 1, 2 and 5 decreased slowly compared with other intersections. But the average traffic volume increased significantly at the south import of intersection 3, north import of intersection 4, east and north imports of intersection 7. These phenomena show that when parking lots are saturated, parking lots act as traffic attraction points, resulting in increased traffic pressure at intersections which with a high correlation to parking lots.

**Figure 7:**
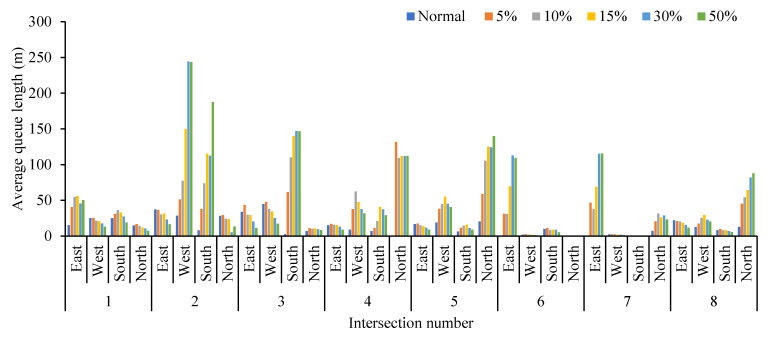
Comparison of average queue length at different intersections.

**Figure 8:**
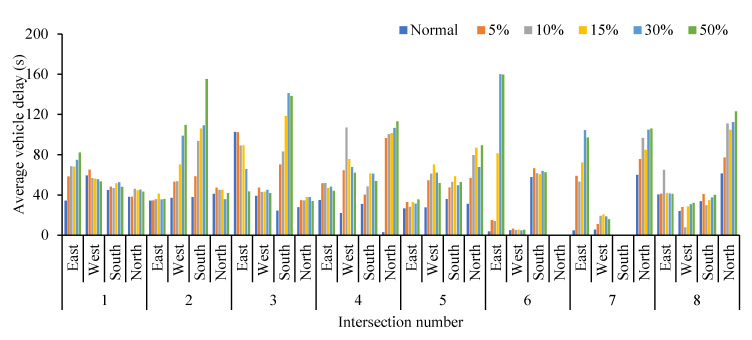
Comparison of average vehicle delay at different intersections.

**Figure 9:**
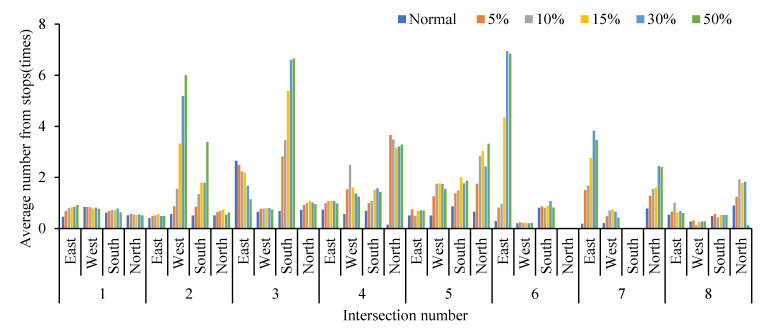
Comparison of average number form stops at different intersections.

Obviously, the average queue length, the maximum queue length, the average vehicle delay and the average number form stops all increase with the increase in the proportion of cruising vehicles, which are positively correlated. Conversely, the average traffic volume drops significantly. When the proportion of cruising vehicles grows at 15%, the average queue length at the intersection increases significantly, from 37.58 m to 45.90 m. When the proportion of cruising vehicles reaches 50%, the average queue length increases by 49.82 m. It is not convenient for cruisers to change lanes before entering the intersection, particularly confronting a long queue length. This inconvenience aggravates traffic congestion at the intersections. From Donald Shoup’s observation in Los Angeles, the cruising vehicles mixing in the normal traffic flow accounted for 30% [1]. Therefore, the simulation of 30% mixed traffic flow was taken to exemplify the influence of cruising for parking on the traffic efficiency at intersections. The average queue length, maximum queue length, average traffic volume, average vehicle delay and average number form stops at the intersections under 30% mixed traffic flow increases by 235.1%, 143.2%, −24.42%, 92.0% and 192.1%, respectively, compared with normal traffic flow. According to the standard of level of service at signalized intersections [30], the average LOS of normal traffic flow is Level C, while 30% of mixed traffic flow is Level D. Therefore, cruising for parking behavior has a negative impact on the intersection. Take the average queue length, average vehicle delay, and average number form stops for examples, as shown in Figure 7, Figure 8 and Figure 9. It can be seen that the influence degree of 8 intersections is different. It is more significantly obvious on intersections 2, 3, 4, 6 and 8, which indicated that the intersection higher the correlation with parking lots, the more influenced by cruising traffic flow. Therefore, it is important to optimize parking facilities and improve the utilization rate of parking spaces to alleviate the impact of cruising for parking behavior on traffic flow.

**Figure 10:**
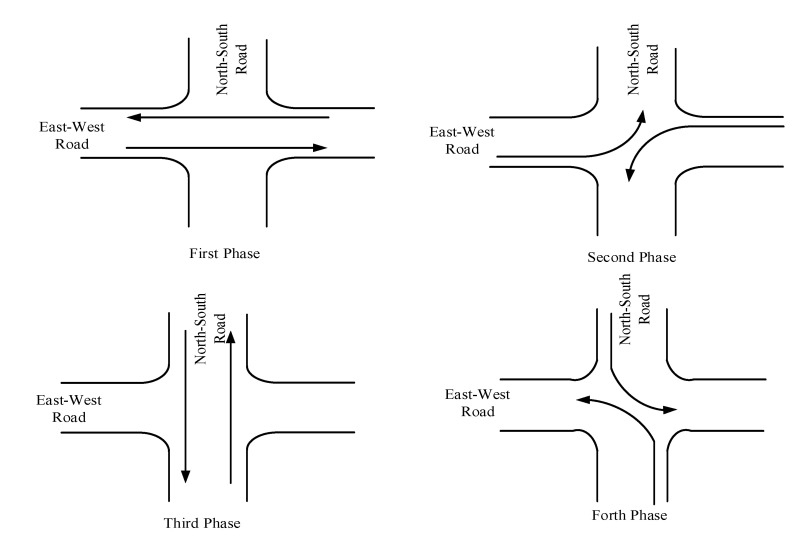
The traffic phase diagram at No. 3 intersection.

**Figure 11:**
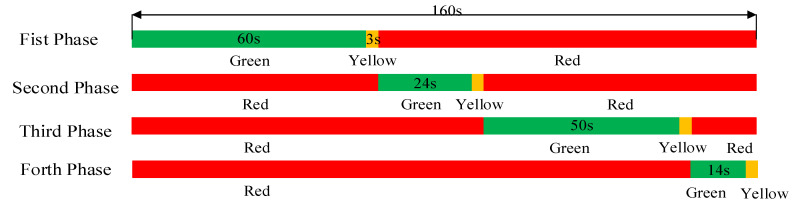
Phase timing diagram at No. 3 intersection.

**Figure 12:**
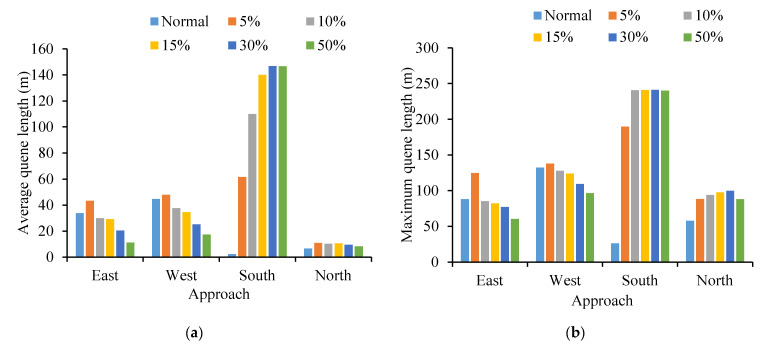

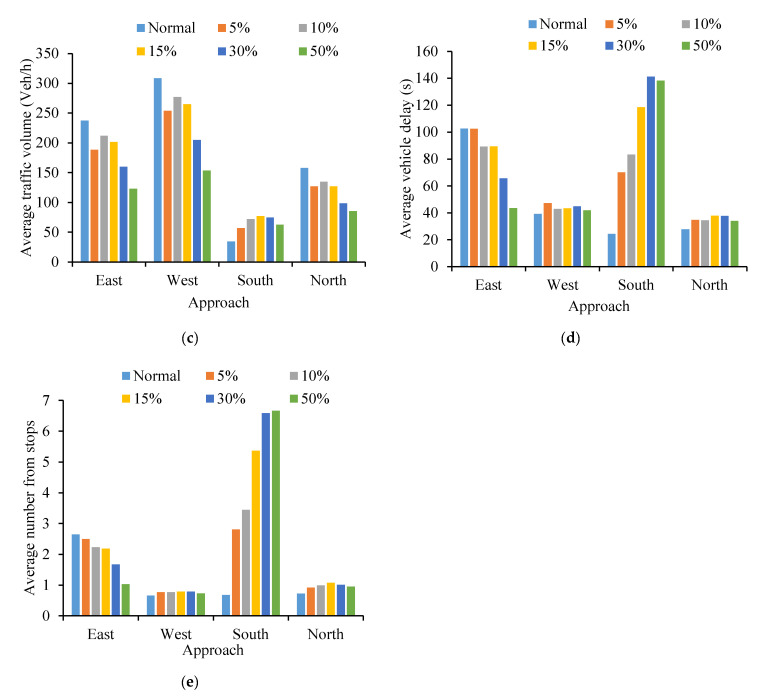
Comparison of five MOEs at different approaches of no. 3 intersection. (**a**) Average queue length at different approaches of no. 3 intersection. (**b**) Maximum queue length at different approaches of no. 3 intersection. (**c**) Average traffic volume at different approaches of no. 3 intersection. (**d**) Average vechicle delay at different approaches of no. 3 intersection. (**e**) Average number from stops at different approaches of no. 3 intersection.

Taking four approaches of intersection 3 for example, the traffic phase diagram and phase timing diagram are shown as Figure 10 and Figure 11. Five MOEs are compared in detail under the different proportions of cruising vehicles. As shown in Figure 12, it can be seen that the four MOEs (except the average traffic volume) of the south approach of intersection 3 increase with the increase in cruising proportion. However, the average traffic volume decreases with the increase in cruising proportion after reaching the saturated flow of traffic capacity. In addition, the negative impact of cruising for parking behavior on this approach is greater than others. Due to the direct link between the south approach and parking lot no. ②, it is the entrance of the intersection where cruising vehicles pass through parking lot No. ②, while other approaches are not. Therefore, it is proved that the intersection with a higher degree of correlation with the parking lot is more affected by cruising behavior.

#### 5.1.2. Influence of Different Cruising Ratios on Traffic Flow on the Road Sections

**Figure 13:**
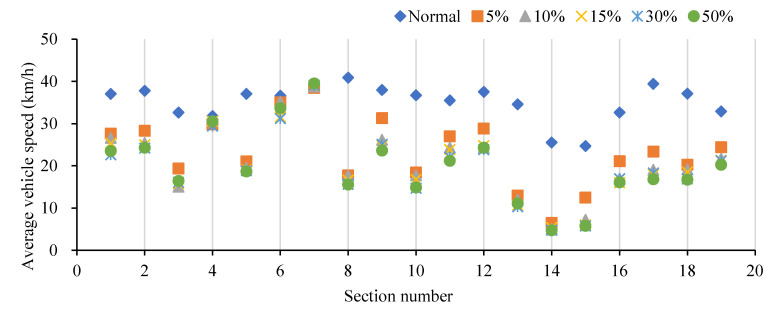
Speed distributions on the road sections under different cruising ratios.

Similarly, the speed distribution was chosen as the indicator of road sections. The speed distributions of 0%, 5%, 10%, 15%, 30% and 50% cruising traffic flows on the road sections are shown in Figure 13. From the overall perspective, the average speed of road section are 35.21 km/h, 23.6 km/h, 21.4 km/h, 20.6 km/h, 20.1 km/h and 20.0 km/h, respectively, and the speed decreases with the increase in the proportion of cruising vehicles. From a local point of view, the speed distribution of each road section is negatively correlated with the proportion of cruising vehicles. Each detection point (as a detection point corresponds to one lane, which is referred to as the lane in the following text) was taken as an object of analysis separately. It analyzes that the increase in the proportion of cruising vehicles for parking had no significant impact on the speed of some specified lanes. For example, approximately 10 lanes such as no. 1 and no. 8 have low correlations with parking lots, and the average speeds are keeping around 40 km/h. However, the other 25 lanes such as no. 2 and no. 34 have a significant impact on the speed. Due to their high correlation with parking lots, the maximum growth rate of the mixed traffic flow between 5% and 50% in the same lane is −863.74%. Therefore, cruising for parking behavior has a negative impact on traffic efficiency of the road section.

**Figure 14:**
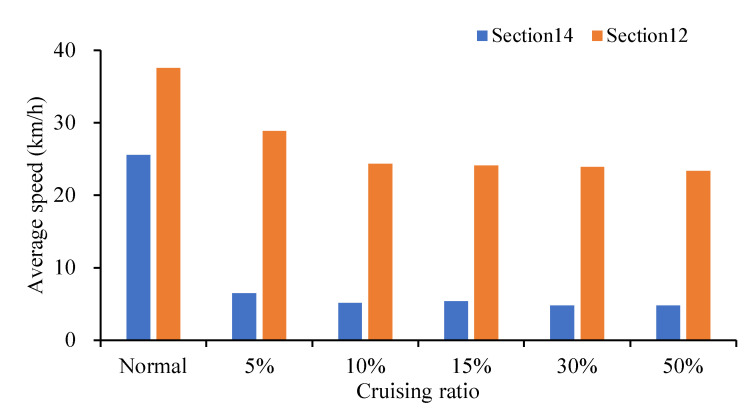
Comparison of average speed at Section 12 and Section 14.

Sections 12 and 14 were also chosen as an example for comparing the impact of cruising on the different sections. As shown in the Figure 14, the average speed decreases with the increase in cruising ratio (from 5%, 10%, 15%, 30% to 50%) by 74.58%, 79.88%, 79.00%, 81.33% and 81.36%, respectively. It can be seen that cruising for parking behavior has a significant impact on this section, and the speed reduction reached 74.58% when the cruising ratio is 5%. Section 12 was selected as a basic scenario and control group for speed comparison. As shown in Figure 14, the average speed decreases with the increase in cruising ratio, decreasing by 23.20%, 35.25%, 35.80%, 36.39% and 37.90%, respectively. It can be seen that cruising for parking behavior has a negative impact on this section. It is not as serious as Section 14. The speed reduction is all lower than Section 14. Since there is no parking entrance or exit at Section 14, it is directly related to parking lot no. ①. It is 307 m from parking lot no. ①. While there is no entrance or exit of the parking lot at Section 12 as well, which is not directly related to parking lot no. ①, 822 m away from parking lot no. ①. Therefore, there is so much difference. The cruising vehicles mostly drive in Section 14, with a high correlation and closer distance to the parking lot.

#### 5.1.3. Influence of Different Cruising Ratios on Traffic Flow of Road Network

**Figure 15:**
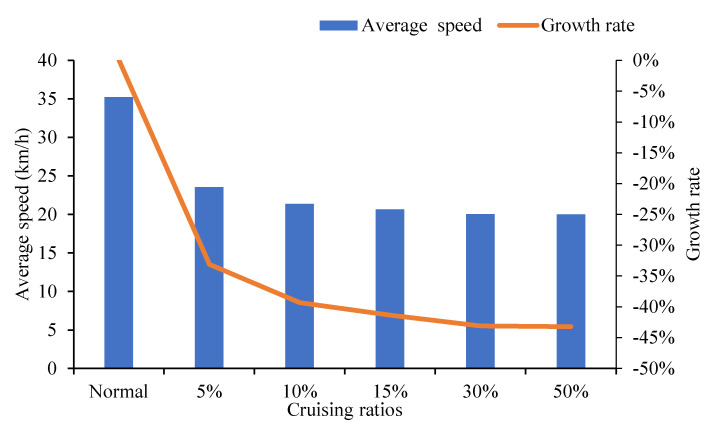
Comparison of average speed at road network.

It can be concluded from the influential analysis on the road sections and intersections that the average speed of road network decreases with the increase in the proportion of cruising vehicles. As shown in Figure 15 when the proportion of cruising vehicles are 5% and 10%, the speed drops significantly, by 33.12% and 6.19%, respectively, compared with the normal traffic flow scenario. The influence of cruising behavior begins to weaken when the proportion of cruising vehicles is 15%. When the ratio is 30%, the capacity of road network reaches a saturated threshold, and the declining trend of the average speed slows down. When the proportion reaches 50%, it only decreases by 0.15% from 30% to 50% cruising ratio.

**Figure 16:**
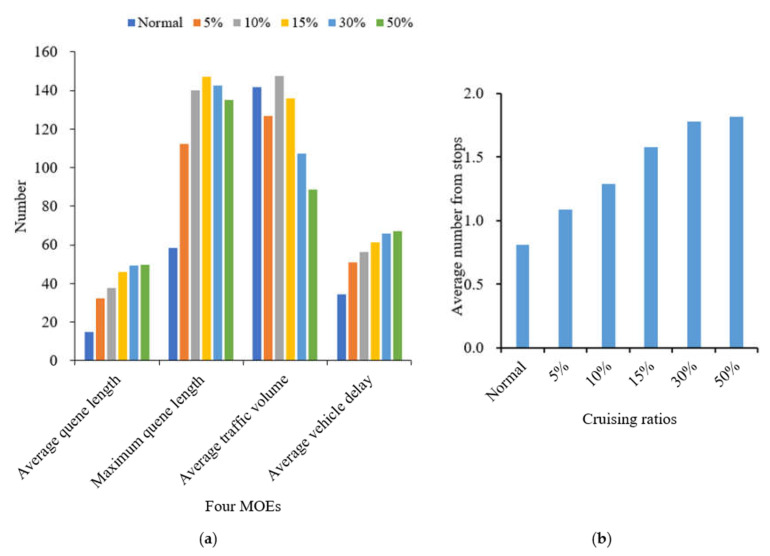
Comparison of five MOEs at road network. (**a**) Four MOEs at road network. (**b**) Average number from stops at road network.

**Figure 17:**
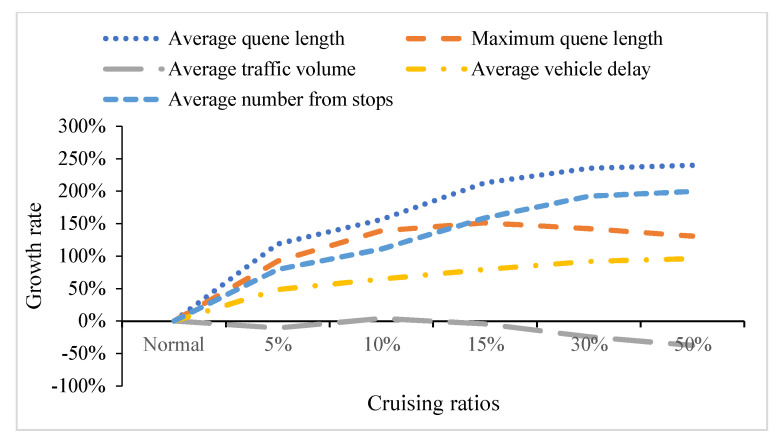
Five MOEs’ growth rate.

Similarly to the influence analyzed previously, four MOEs grow in general, while the average traffic volume drops on the contrary in Figure 16 and Figure 17. When the proportion of mixed traffic flow reaches 5%, the queue length surges. When the proportion climbs up 30%, the average traffic volume on the road network reaches its capacity. It is impossible to enter new vehicles due to serious traffic congestion. The growth of average number form stops, average queue length, maximum queue length and average vehicle delay tends to be flat, and the average traffic volume at the intersection begins to decrease. Therefore, cruising behavior also has a negative impact on the road network.

Combining the average speeds of the various sections and the average vehicle delay, average queue length, maximum queen length and average number form stops of frequent traffic jams, traffic performance index (TPI) for road network is weighted and calculated for different cruising ratios, 5% for 2–4 and 30% for 4–6. TPI describes road network performance deteriorating with the increase in the cruising ratio. The traffic flow status on the network changes from light to severe congestion. It is proving that cruising for parking is an invisible contributor to traffic congestion. So it is necessary for traffic managers and engineers to pay close attention to the cruising behavior to ease traffic congestion.

### 5.2. Influence of Cruising for Parking Behavior on Traffic Emissions

The variety of emissions (CO, NO_X_, VOC) and fuel consumption under the mixed cruising traffic flow were selected to analyze the impact of cruising for parking on the traffic emission on the road section, intersection, and network. As shown in Table 4, the average total emissions of the road network straight up with the increase in the proportion of cruising vehicles. When the proportions of cruising vehicles are 5% and 10%, the total emission increases significantly, increasing by 32.52% and 67.77%, respectively. While the proportion reaches 15%, the influence of cruising behavior begins to weaken. When the proportion is 30%, the road network carrying capacity reaches a saturated threshold and the growth trend of the average total emissions slows down. When the proportion reaches 50%, the emissions only increase by 3.35% compared with the proportion of 30%. Therefore, cruising for parking behavior increases traffic emissions. It is recommended to reduce cruising for parking behavior through reasonable traffic organization and management strategies to alleviate serious traffic emission. This is the key to creating a good traffic environment.

#### 5.2.1. Influence of Different Cruising Ratios on Traffic Emissions at the Intersections

**Figure 18:**
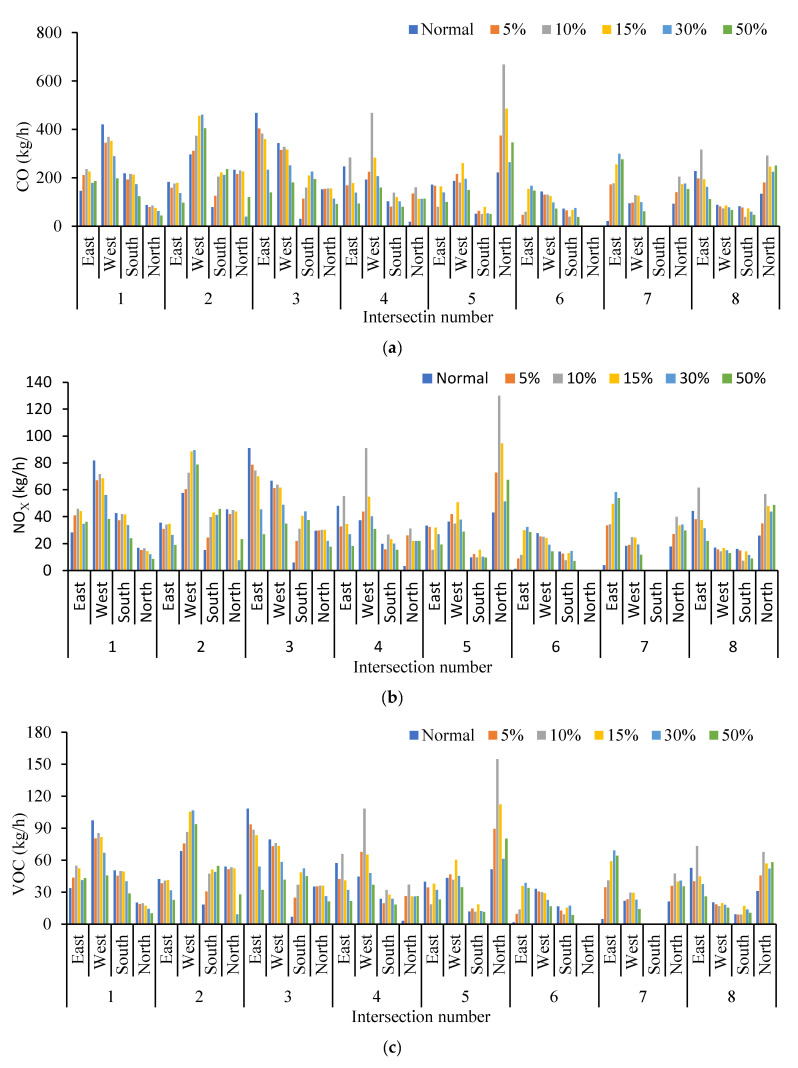

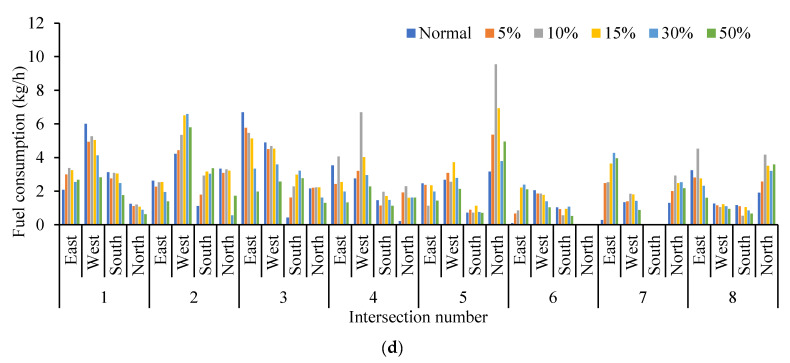
Comparison of four indicators at different intersections. (**a**) Comparison of CO emission at different intersections. (**b**) Comparison of NO_X_ emission at different intersections. (**c**) Comparison of VOC emission at different intersections. (**d**) Comparison of fuel consumption at different intersections.

The four indicators of traffic emissions at intersections with different traffic flows were compared in Figure 18. The distribution trends of the four indicators are similar. Because most cruising for parking vehicles driving back and forth around a parking lot form a relatively fixed search path, the four indicators at west and south imports of intersection 2, south import of intersection 3, north import of intersection 5, east import of intersections 6 and 7, and north import of intersection 8 are increased with the increase in the cruising vehicles. These entrances are the only entrances and exits for cruising vehicles to enter the parking lots. In order to find parking spaces, cruisers frequently drive at these entrances, resulting in additional emissions and fuel consumption. Meanwhile, the fixed concentration of vehicle flow lead to the reduction in emissions and fuel consumption at other intersections, but the overall emissions and fuel consumption increased.

**Figure 19:**
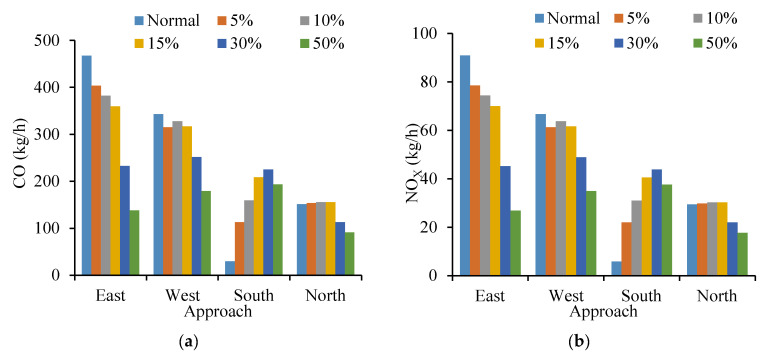

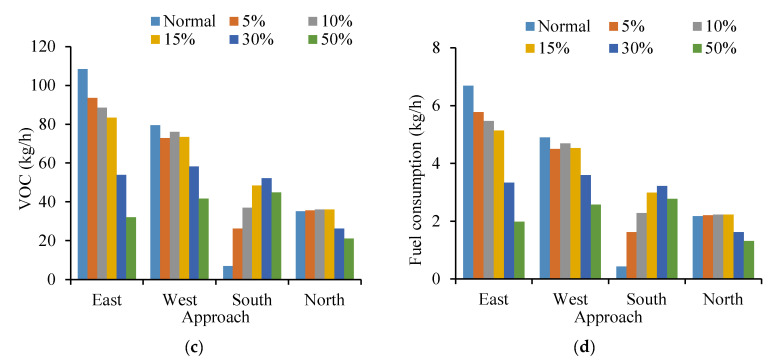
Comparison of No. 3 intersection emission. (**a**) Comparison of CO emission under different approaches at No. 3 intersection. (**b**) Comparison of NO_X_ emission under different approaches at No. 3 intersection. (**c**) Comparison of VOC emission under different approaches at No. 3 intersection. (**d**) Comparison of fuel consumption under different approaches at No. 3 intersection.

As shown in Figure 19, intersection no. 3 was also selected as the typical example. It can be seen that all indicators (CO, NO_X_, VOC and fuel consumption) for the south approach of the intersection increase with the increase in the cruising vehicles. When the proportion of cruising reaches 50%, the traffic capacity of the intersection drops slightly, and the negative impact of cruising for parking on south approach is greater than others. That is because the south approach is directly related to parking lot no. ② and it is the necessary entrance for cruising vehicles passing through parking lot no. ②. Contrary to other approaches, it indicates that the cruising vehicles frequently drive at the intersection approach, which is closely connected with the parking lot.

#### 5.2.2. Influence of Different Cruising Ratios on Traffic Emissions at the Sections

**Figure 20:**
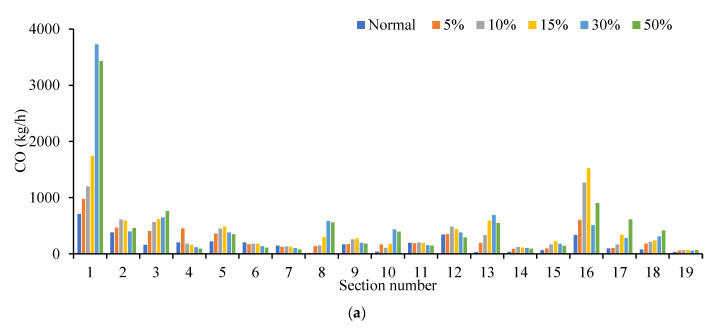

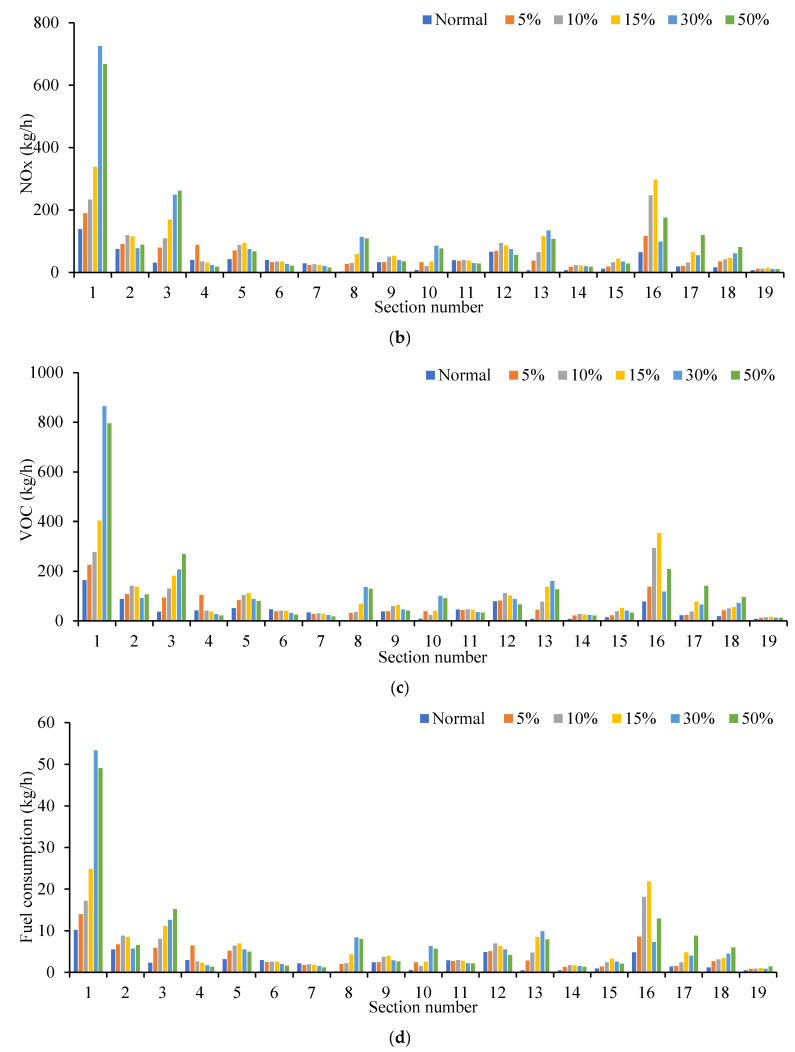
Comparison of four indicators at different sections. (**a**) Comparison of CO emission under different sections. (**b**) Comparison of NO_X_ emission under different sections. (**c**) Comparison of VOC emission under different sections. (**d**) Comparison of fuel consumption under different sections.

The four indicators of traffic emission at intersections with different traffic flows were compared in Figure 20. The distribution trends of the four indicators are similar. Four indicators at sections 1, 3, 8, 10, 13, 15, 17 and 18 all increased with the increase in cruising vehicles. Cruisers on the road for a short tour parking choice decisions, frequent lane changing and deceleration or lower speed observation, cause emissions and fuel consumption increase, affect other vehicles on the road at the same time. As a result, the emissions of this section were higher than that of surrounding sections.

**Figure 21:**
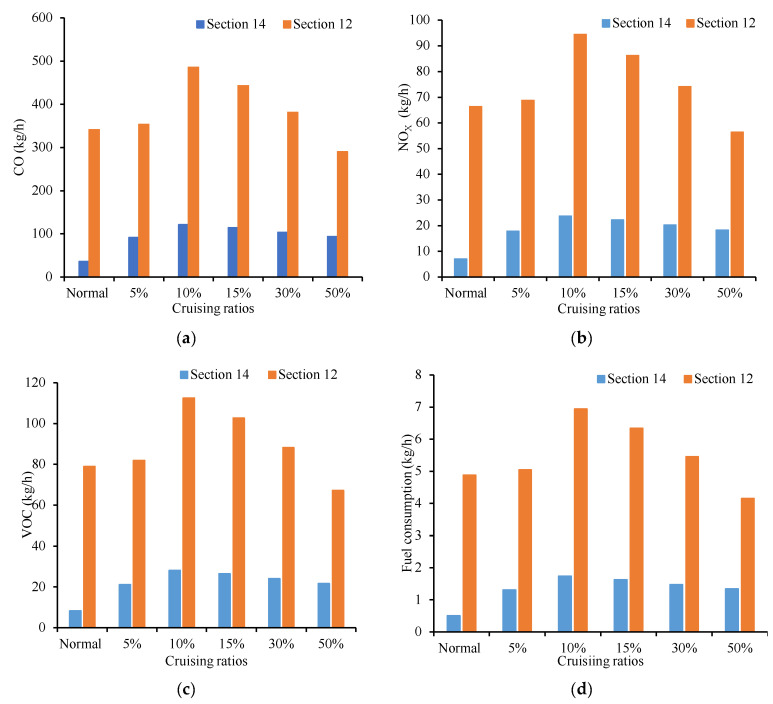
Comparison of Section 14 and Section 12 emission. (**a**) Comparison of CO emission under Section 14 and Section 12. (**b**) Comparison of NO_X_ emission under Section 14 and Section 12. (**c**) Comparison of VOC emission under Section 14 and Section 12. (**d**) Comparison of fuel consumption under Section 14 and Section 12.

Likewise, Figure 21 shows that all the four indicators for road sections increase with the growth of the cruising flow. The total emissions of Section 14 increases by 155.16%, 238.97%, 238.97%, 250.03%, 259.03% and 265.69% according to the proportion of cruising flow (5%, 10%, 15%, 30% and 50%). It can be seen that the behavior of cruising for parking has a significant impact on the emission of this section. The emission growth rate reaches 155.16%, even though the cruising ratio is only 5%.

Correspondingly, the emissions of Section 12 increases by 3.67%, 42.41%, 43.16%, 44.76%, and 48.14% as the proportion of cruising rises. They are all lower than Section 14. Although the behavior of cruising for parking also has a negative impact on this section, its influence is less than Section 14. The reason for this distinction is the different locations of Section 14 and Section 12. There is no parking lot entrance and exit at Section 14, but it is directly related to parking lot no. ①. However, there is no entrance-exit of parking lot at Section 12, which is not directly related to parking lot no. ①. It is away from parking lot no. ① by 822 m. It proves that the section, which with higher relevance to the parking lot is more affected by cruising for parking behavior than others.

#### 5.2.3. Influence of Different Cruising Ratios on Traffic Emissions on the Road Network

**Figure 22:**
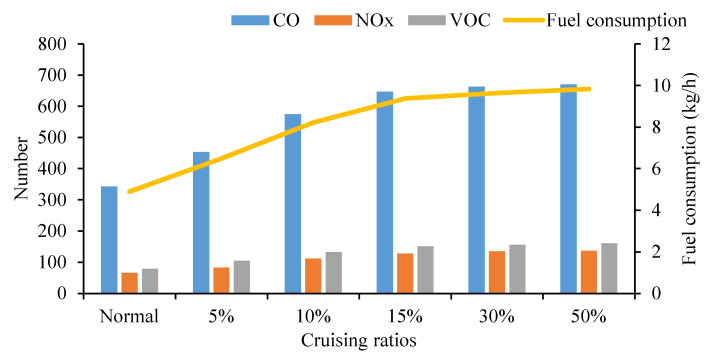
Traffic emissions on the road network.

As shown in Figure 22, the total emissions increase significantly when the cruising ratios are 5% and 10%, which are an increase of 32.52% and 67.77%, respectively, compared with the normal traffic flow. While the proportion of cruising ratio is 15%, the influence of cruising behavior begins to weaken. When the cruising ratio is 30%, the road network’s traffic flow reaches its carrying capacity, and the growth trend of the emissions slows down. For the 50% cruising proportion, it only increases by 3.35% compared with the 30% proportion.

**Figure 23:**
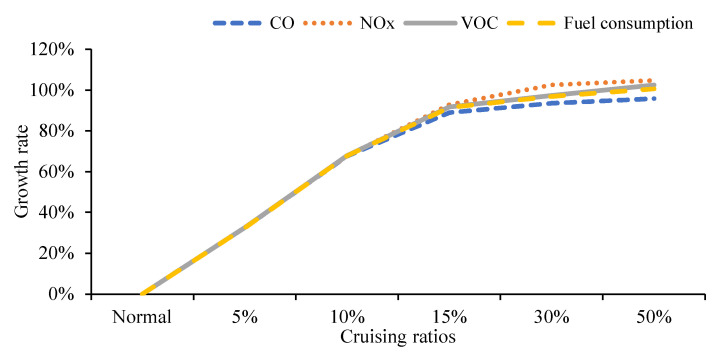
Growth rate of road network emissions.

As shown in Figure 23, emissions of CO, NO_X_ VOC and fuel consumption all increase as the proportion of cruising vehicles increases. When the proportion of cruising cars reaches 15%, the tendency of the growth tends to be flat. Cruising behavior has a negative impact on the overall road network.

## 6. Layout Optimization of Parking Lots Based on Minimizing Cruising Distance

**Figure 24:**
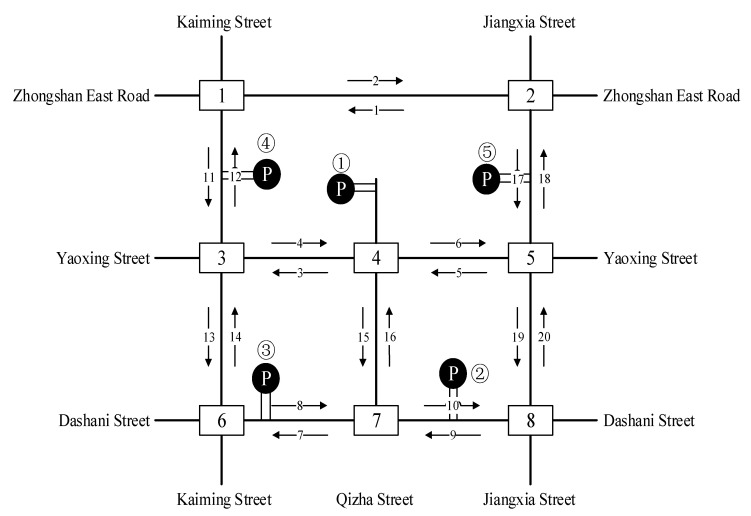
Comparison of parking location schemes.

It can be concluded from the influential analysis of Section 5, the closer distances the intersections and sections are to the parking lot, the greater the impact is. Therefore, the cruising distance is reduced by changing the location and access road of the parking lot to minimize the influential effects. The current parking lots’ location and road access are shown in Figure 24. Taking parking lot no. ① as an example, it is surrounded by large commercial and recreational buildings and has the highest parking demand in the CBD. Its access roads are located on the approaches to the Qizha and Yaoxing intersection. The main problems of current parking lot locations include: (a). Too close to the intersections; (b). Direct links among the five parking lots are much fewer to increase the cruising distances; (c). Access roads are set up near the approaches with a high level of traffic flow.

For details of negative impacts, parking lots no. ② and ③ are too close to the adjacent intersections and obstruct the diversion of the entrance and exit lanes at the intersections. So do parking lots no. ① and ④, they are also stuck the arterial roads of Yaoxing Streets. The access to parking lot no. ③ is located on the sidewalk and sorely threatens pedestrians’ safety. Overall, the connectivity of five parking lots is isolated on the road network. So it is difficult for cruising vehicles to search a vacant parking lot and increase detour distance. Sensitivity analysis under different locations and access roads of parking lots is applied for the optimization scheme while the simulation framework keeps other variables at the same level. The optimal scheme is shown in Figure 24. The cruising vehicles could successively reach the remaining parking lots without repeated routes, to achieve path optimization, and effectively improve the accessibility and utilization of each parking lot.

**Figure 25:**
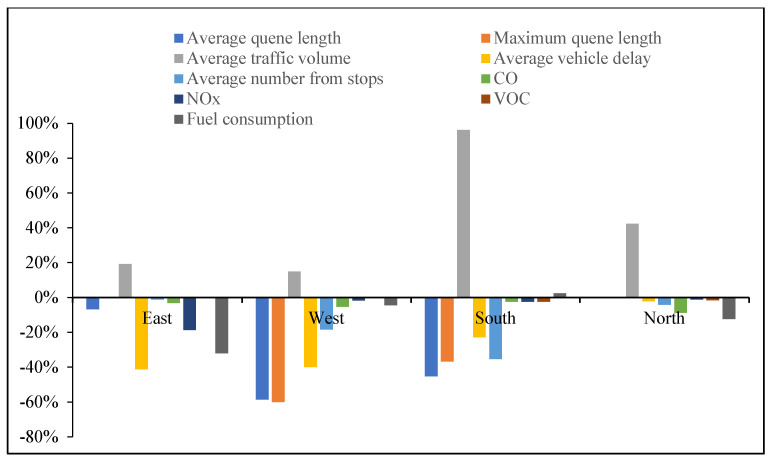
Optimization comparison of No. 5 intersection.

Comparison of the optimal scheme with the current locations of parking lots, the queue length, vehicle delay, and traffic emissions (CO, NO_X_, VOC, and Fuel consumption) significantly decreased in Table 5. In order to further analyze the optimization degree of access roads, a representative parking lot no. ⑤ was selected and its corresponding intersection is no. 5. As shown in Figure 25, the west approach at intersection no. 5 is significantly improved with average queue length shortened by 58.7%, traffic flow increased by 14.9%, average vehicle delay reduced by 40.1%, number form stops reduced by 18.5%, total emissions and fuel consumption reduced by 5.3%, 1.7%, 0.05% and 4.6%, respectively. Originally, the left-turn lane of west approach and the through-lane of south approach are the main routes for cruising vehicles to enter parking lot no. ⑤. After the parking location optimization, the cruising flow decreases. At the same time, fewer cruising vehicles make U-turns to enter the parking lot from the north approach, which reduces the obstruction of normal traffic flow and increases the traffic capacity of the north approach leading drivers to choose the shortest route for cruising for parking.

**Figure 26:**
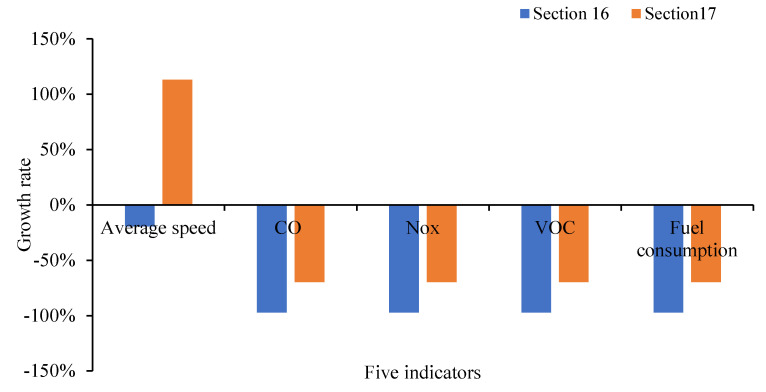
Optimization comparisons at Section 16 and Section 17.

Similarly, the traffic efficiency and emissions for the road sections are improved dramatically by the optimal scheme in Table 6. The results before and after optimization of corresponding sections 16 and 17 relating to parking lot no. ⑤ are compared in Figure 26. The speed of Section 16 is reduced by 19.4%, and CO, NO_x_, VOC and fuel consumption were all reduced by 97.4%. Because the entrance and exit of the parking lot no. ⑤ relocated to Section 16, the overall speed of the section is reduced by the increase in cruising vehicles, while the total emissions were still reduced. The average speed of Section 17 increased by 113.4%, and CO, NO_X_, VOC and fuel consumption all decreased by 69.7%. The overall influence of Section 17 is weakened. Combined with the influence of Section 16, the overall traffic condition is improved, indicating that the optimal location was feasible.

**Figure 27:**
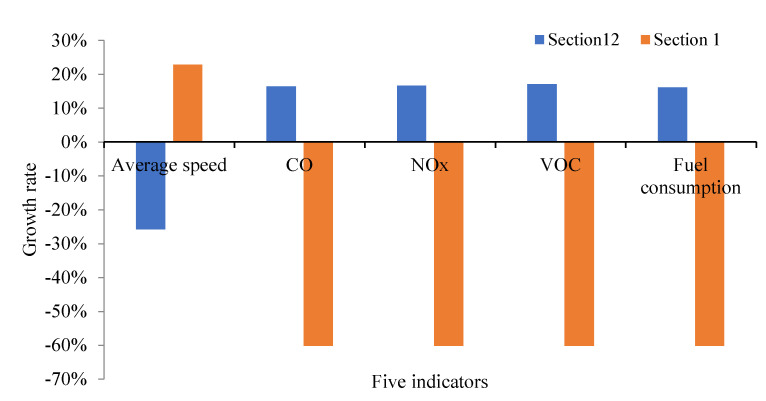
Optimization comparison at Section 12 and Section 1.

Furthermore, the results at sections 1 and 12 before and after optimization of parking lot no. ④ also significantly reduce the negative impact of cruising for parking behavior on the road network in Figure 27.

It can be seen that average queue length, maximum queue length, average traffic volume, average vehicle delay, average number form stops, average speed and total emission at the sections and intersections are significantly improved compared with the current situation. Therefore, it can be concluded that the improved scheme has a significant effect on reducing the negative impact of cruising behavior.

## 7. Discussion and Conclusions

In this study, the parking-and-visit cruising tests with GPS and cameras were applied to collect the behavior of the cruisers, and the videotapes of traffic flows were used to measure the volume of cruising cars and the traffic status of normal cars, simultaneously. The simulation framework based on the microscopic VISSIM was proposed for reproducing the cruising vehicles and normal traffic flows. The car-following model of cruising vehicles was calibrated by GPS and video data. The scenarios under different cruising vehicle ratios were analyzed to quantitatively evaluate the influence of cruising for parking on the traffic efficiency and emission on the road section, intersection and network, respectively. Finally, the layout optimization changing the parking locations and positions of entrance-exit gates were discussed for minimizing the cruising distance and mitigating the negative effect. The results show that: (1) With the increase in the proportion of cruising vehicles, the average queue length, maximum queue length, average vehicle delay, average number from stops and total emissions all increase, showing a positive correlation, while the average speed and traffic volume are on the contrary. (2) The negative impact on the intersections is related to the connectivity degree with parking lot. The closer the distance is to the parking lot, the greater the negative effect is to the corresponding approach of the intersection. Similarly, the road sections accessing with entrance and exit of parking lots are significantly affected more than others. (3) The average speed of road network decreases with the increase in the proportion of cruising vehicles, and the average queue length, maximum queue length, vehicle delay, average number from stops and average total emissions all increase. The impact of cruising for parking behavior begins to slow down after the ratio is 15%. When the ratio reaches 30%, traffic efficiency on the road network reaches the carrying capacity. Traffic congestion is too serious, so the new vehicles can no longer enter the road network, and the hourly volume of the road network begins to decrease. The impact of cruising for parking deteriorates the LOS of traffic performance on the road network. (4) The cruising distance is reduced through changing the location and access road of the parking lot to minimize the influential effects. Sensitivity analysis results indicated that the entrance and exit accessing road network are set on the branch road for improving the connectivity of the network and allocating traffic pressure, which effectively mitigates the negative impact. The results provide a quantitative method for the hidden contribution of cruising for parking to traffic congestion and emission, and provide a new perspective for the improvement of traffic operation efficiency and traffic emission control. The quantitative influence of cruising for parking will be reliably evaluated for the age of AVs.

Additionally, the proposed microscopic perspective is beneficial to develop the optimal strategies to eliminate the influence of cruising for parking on traffic performance. The specific parking policies such as dynamic parking pricing, parking space reservation and intelligent parking guidance can be discussed and assembled into the proposed model. This work will be extended in future studies to evaluate the cruising for parking behavior of AVs.

## Figures and Tables

**Table 1:** Characteristics of Test Sections.

Test Section	Minimum	Maximum	Mean	Standard Deviation
Search time (min)	1.350	29.710	6.030	1.964
Speed of cruising car (km/h)	0.100	52.130	13.530	7.586
Acceleration of cruising car (m/s^2^)	0.010	0.530	0.250	0.281
Number of acceleration of cruising car (times)	9.000	45.000	27.410	2.878
Number of lane-change in cruising car (times)	0.000	17.000	4.790	1.893
Frequency of lane-change in cruising car	0.100	0.790	0.370	0.032
Distracted time of cruising driver (s)	0.000	19.000	3.530	4.754

**Table 2:** Widemann74 car-following model parameters.

Parameter	Meaning	Value	Minimum	Maximum	Parameter	Meaning	Value
*L_n_*_−1_	Front vehicle captain	4.5 m	-	-	b_min_	Maximum deceleration	−2.5 m/s^2^
*v_des_*	Road speed limit	60 km/h	-	-	OPDV_add_	Expected speed difference and coefficient	1.5
*ax_add_*	Expected stopping distance and coefficient	1.25	1.0 m	2.5 m	OPDV_mult_	Expected speed difference multiplication factor	1.5
*ax_mult_*	Expected stopping spaces multiplication factor	2.5			cx	Velocity difference parameter	40
*bx_add_*	Expected car following distance and coefficient	4.0	1.0 m	5.0 m	NRND	Random Variable	(0, 1)
*bx_mult_*	Expected car following distance multiplication factor	3.0	2.0 m	6.0 m	RND1	Random Variable	(0, 1)
*ex_add_*	Expected spacing multiplication factor	1.5	-	-	RND2	Random Variable	(0, 1)
*ex_mult_*	Expected spacing multiplication factor	0.55	-	-	RND3	Random Variable	(0, 1)
*BNULL_add_*	Acceleration/deceleration multiplication factor	0.1	-	-	RND4	Random Variable	(0, 1)
*b_max_*	Maximum acceleration	5 m/s^2^	-	-			

**Table 3:** Traffic flow status at the intersection.

Proportion of the Cruising Vehicles	Average Queue Length (m)	Maximum Queue Length (m)	Average Traffic Volume (Veh/h)	Average Vehicle Delay (s)	Average Number Form Stops (Times)
0%	14.66	58.53	141.82	34.21	0.61
5%	32.09	112.45	126.63	50.78	1.09
10%	37.58	139.95	147.51	56.29	1.29
15%	45.90	146.99	135.67	61.38	1.58
30%	49.12	142.35	107.19	65.68	1.78
50%	49.82	135.13	88.48	67.09	1.82

**Table 4:** Average emissions and fuel consumptions under different cruising ratios.

Types	CO (kg/h)	NO_X_ (kg/h)	VOC (kg/h)	Fuel Consumption (kg/h)
Normal traffic flow	342.49	66.63	79.33	4.90
5% mixed traffic flow	453.87	88.31	105.19	6.49
10% mixed traffic flow	574.59	111.79	133.17	8.22
15% mixed traffic flow	647.15	128.53	152.10	9.38
30% mixed traffic flow	662.58	135.34	156.58	9.65
50% mixed traffic flow	670.66	136.84	160.63	9.83

**Table 5:** Comparison results at the intersections.

Types	Average Queue Length (m)	Maximum Queue Length (m)	Average Traffic Volume (Veh/h)	Average Vehicle Delay (s)	Average Number Form Stops (Times)	CO (kg/h)	NO_X_ (kg/h)	VOC (kg/h)	Fuel Consumption (kg/h)
30% mixed traffic flow (before)	49.1	142.4	107.2	65.7	1.8	167.3	32.6	38.8	2.4
30%mixed traffic flow (after)	31.1	106.5	125.7	61.0	1.7	115.6	32.0	38.0	2.1

**Table 6:** Comparison of results of the sections.

Types	Average Speed (km/h)	CO (kg/h)	NO_X_ (kg/h)	VOC (kg/h)	Fuel Consumption (kg/h)
30% mixed traffic flow (before)	19.9	495.2	102.8	117.8	7.3
30% mixed traffic flow (after)	25.2	470.4	91.5	109.0	6.7

## Data Availability

The data used to support the findings of this study are available from the corresponding author upon request.
